# Natural protein sequences are more intrinsically disordered than random sequences

**DOI:** 10.1007/s00018-016-2138-9

**Published:** 2016-01-22

**Authors:** Jia-Feng Yu, Zanxia Cao, Yuedong Yang, Chun-Ling Wang, Zhen-Dong Su, Ya-Wei Zhao, Ji-Hua Wang, Yaoqi Zhou

**Affiliations:** Shandong Provincial Key Laboratory of Biophysics, Institute of Biophysics, Dezhou University, Dezhou, 253023 China; College of Physics and Electronic Information, Dezhou University, Dezhou, 253023 China; Institute for Glycomics and School of Information and Communication Technology, Griffith University, Parklands Dr, Southport, QLD 4222 Australia

**Keywords:** Random sequence, Protein intrinsic disorder, Secondary structure, Molten globule, Molecular dynamics simulation

## Abstract

Most natural protein sequences have resulted from millions or even billions of years of evolution. How they differ from random sequences is not fully understood. Previous computational and experimental studies of random proteins generated from noncoding regions yielded inclusive results due to species-dependent codon biases and GC contents. Here, we approach this problem by investigating 10,000 sequences randomized at the amino acid level. Using well-established predictors for protein intrinsic disorder, we found that natural sequences have more long disordered regions than random sequences, even when random and natural sequences have the same overall composition of amino acid residues. We also showed that random sequences are as structured as natural sequences according to contents and length distributions of predicted secondary structure, although the structures from random sequences may be in a molten globular-like state, according to molecular dynamics simulations. The bias of natural sequences toward more intrinsic disorder suggests that natural sequences are created and evolved to avoid protein aggregation and increase functional diversity.

## Background

Proteins are linear polymeric chains made of a combination of 20 different types of amino acid residues. The total number of proteins explored by nature since the origin of life is estimated between 10^21^ and 10^43^ [1]. This number is infinitesimal compared to the number of possible protein sequences because the sizes of proteins can range from 2 to as long as 35,000 amino acid residues [2] and even for a small protein of 100 amino acid residues, the number of possible proteins with distinct sequences is 20^100^ or 10^130^. The tiny sequence space explored by the nature raises an interesting question: if and how random-sequence proteins differ from natural proteins constrained by their functional and structural requirements? Investigating random sequences is also important because some proteins can arise suddenly from non-coding regions [3, 4]. Frame-shifting translation that produces random sequences after the insertion/deletion point was also proposed for the creation of novel proteins [5].

Artificial proteins with random sequences have been studied experimentally. Random co-polymerization of mixed amino-acid* N*-carboxyanhydrides was shown to produce compact structures similar to proteins [6, 7]. Random sequences of three residue types (Q, R, and L) of 70–90 amino acid residues were expressed in *E. coli* and shown to have secondary structures and cooperative unfolding [8]. Further studies indicate that random 120-amino-acid sequences of 20 residue types are aggregation-prone, and 12 residue-type sequences have better solubility [9]. Some soluble proteins are found to be compact with some secondary structures. Chiarabelli et al. [10] showed that 20 % of 79 random 50-residue proteins are likely folded as they were protected from serine protease thrombin. Two of the selected proteins can reversibly fold and unfold. LaBean et al. [11] studied about 30 71-residue random-sequence proteins and found some with high secondary-structure contents with cooperative unfolding. These latest experimental studies suggested frequent appearance of native-like properties in random-sequence proteins. However, the sequences in these studies were obtained according to prescribed frequencies of DNA bases. They may not reflect natural usages of amino acid residues. In addition, codon usage bias in an expression system such as *E. coli* may provide additional biases toward proteins actually expressed. Furthermore, three-dimensional structures of these random-sequence proteins were not determined by either NMR or X-ray crystallography. In fact, other studies suggested the rare occurrence of stably folded or functional proteins. For example, only several functional proteins [12] resulted from initial 4 × 10^12^ random sequences followed by many iterations of in vitro selections and directed evolution [13]. No folded structures were yielded from in vitro random recombination of secondary structure elements (blocks) [14, 15].

Random-sequence proteins were also studied computationally, and two different views emerged. Some supported the view that natural sequences differ only slightly from random sequences [16]. For example, Weiss et al. [17] showed that random protein sequences have similar information content as non-redundant natural protein sequences. Crooks et al. [18] found that protein sequence-structure correlations based on mutual information in sequences of natural proteins can also be generated from random-sequence proteins. Lavelle and Pearson [19] investigated four- and five-amino-acid segments and found no significant biases between natural and random sequences. Angyan et al. [20] compared natural sequences to random protein sequences generated from random DNA sequences at various GC contents. They found that at 40–60 % GC contents, intrinsic disorder and aggregation propensity of translated random proteins are similar to those of natural proteins. By contrast, Pande et al. [21] showed that natural sequences have “pronounced deviations from pure randomness, directed toward minimization of the energy of the three-dimensional structure”. Others supported significant difference between random and natural sequences by developing highly accurate two-state classifiers [22–24]. These computational studies, however, were limited mostly to comparing random-sequence proteins to either fully disordered proteins or fully structured proteins.

This paper presents a comparative study of structure and intrinsic disorder of natural and random-sequence proteins. We compared several structural properties of natural and random protein sequences: predicted intrinsic disorder by IUpred [25] and SPINE-D [26], predicted secondary structures by SPIDER 2 [27], and predicted tertiary structures by SPARKS-X [28]. A few selected model structures were simulated by molecular dynamics simulations. The comparison revealed that natural and random sequences have essentially the same structural properties except that the former have more long disordered regions, likely evolved to avoid detrimental aggregation.

## Results

We constructed three databases of 10,000 protein sequences of 60 amino acid residues at 30 sequence identity cut-off (see Materials and methods). There are natural wild-type sequences (Pnat), random sequences generated according to natural occurrences of amino acid types (Prnd) and random sequences generated according to a fixed occurrence at 5 % for every amino acid type (Preq).

**Fig. 1:**
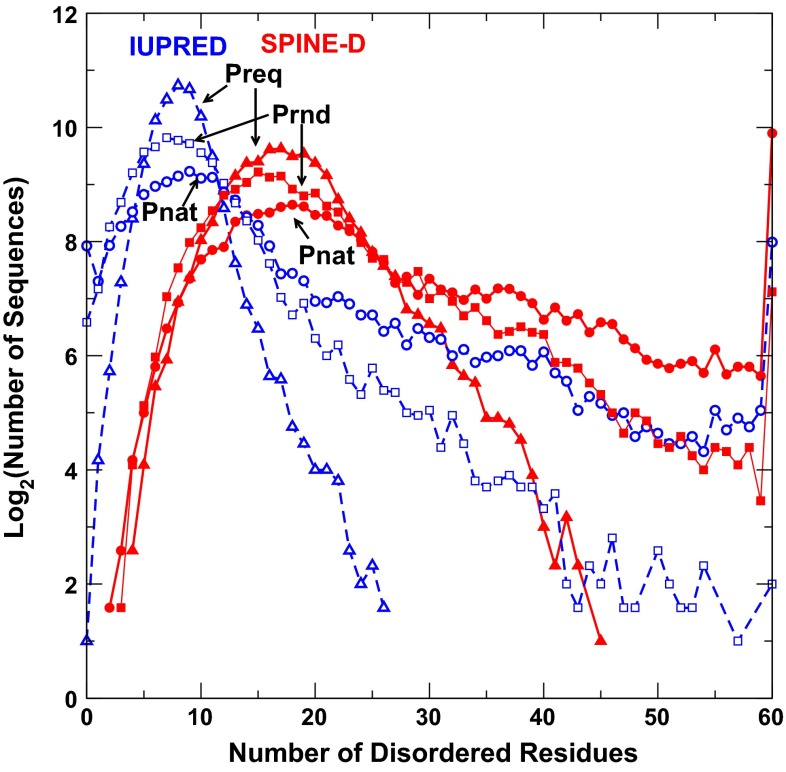
The number of protein sequences (in log2) with a given number of disordered residues predicted by IUpred (in *blue*) and SPINE-D (in *red*) for three separate sequence datasets (natural sequences, Pnat in *circles*; random sequences with natural amino-acid frequencies, Prnd in *squares*; and random sequences with a fixed 5 % frequency for all residues, Preq in *triangles*). Natural sequences are more disordered than random sequences as predicted by either IUPRED or SPINE-D. Here all points with 0 occurrence are not shown

Figure 1 shows the number of protein sequences in number of disordered residues predicted by IUpred and SPINE-D for sequences in Pnat, Prnd, and Preq, respectively. Overall speaking, IUpred predicts more proteins with less number of disordered residues than SPINE-D, regardless of sequence datasets. This observation is consistent with the fact that IUpred has a lower sensitivity than SPINE-D [26]. Nevertheless, IUpred and SPINE-D yield qualitatively similar trends for three sequence databases. That is, natural protein sequences contain less proteins having smaller number of disordered residues (5–26 for SPINE-D) but more proteins having higher number of disordered residues (27–60 for SPINE-D) than random sequences with or without fixing amino-acid compositions at 5 %. The distribution given by random sequences with natural occurrence of amino acid residues (Prnd) is closer to the distribution given by natural sequences (Pnat) rather than to that of random sequences with a fixed composition (Preq). It is of interest to note that natural sequences have more fully disordered proteins (60 residues long) and more fully structured proteins than random sequences although Pnat has only slight more nearly full-structured proteins (number of disordered residues ≤5). Based on SPINE-D, there are 59 natural sequences, 55 random sequences of natural compositions, and 24 random sequences of fixed compositions with ≥55 residues in structured regions. The same trend (more fully structured and more fully disordered proteins for natural sequences) is also observed by IUPRED.

**Fig. 2:**
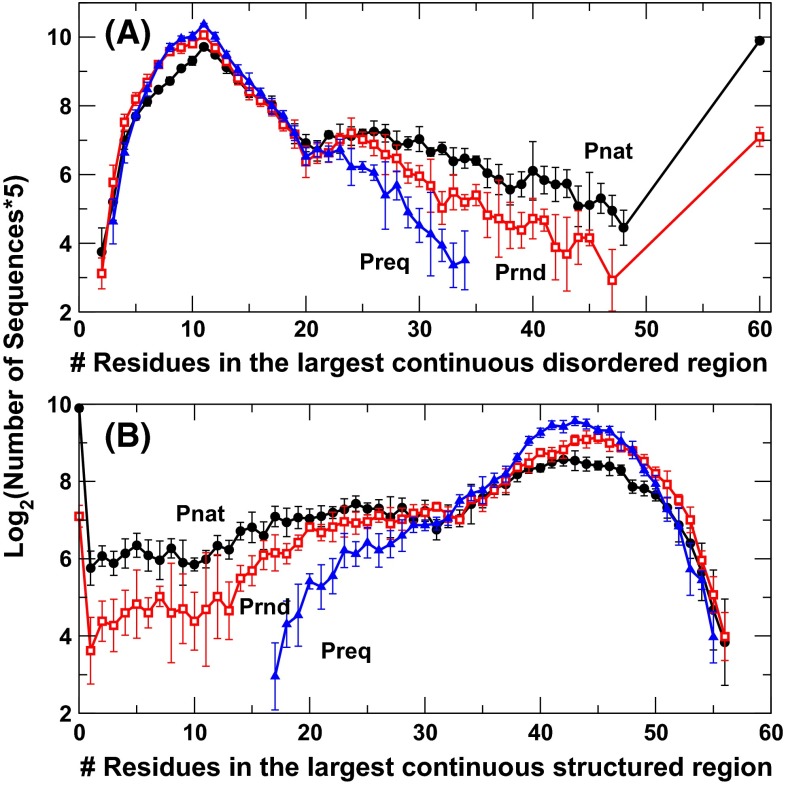
The average number of protein sequences (times 5 in log2) as a function of the number of residues in the largest continuous disordered (**a**) or structured (**b**) regions for three separate sequence datasets (natural sequences, Pnat in *circles*; random sequences with native amino-acid frequencies, Prnd in *squares*; and random sequences with a fixed 5 % frequency for all residues, Preq in *triangles*) according to SPINE-D prediction. 10,000 sequences were randomly divided into five equal subsets. The averages and standard deviations are shown. Natural sequences have slightly more nearly fully structured proteins (>55 residues) than random sequences. Here, all points with 0 occurrence in any subsets are not shown

To confirm that natural sequences have more nearly fully structured and fully disordered proteins, we re-examine the results based on largest continuous disordered or structured regions in Fig. 2. Here we randomly divided 10,000 sequences into five equal sets and obtained the average and standard deviations between five sets of sequences. For clarity, we showed the result from SPINE-D only as IUPRED gives the same trend. Figure 2a indicates that natural sequences have more long disordered regions than sequences in Prnd or Preq. The difference is larger than standard deviation. In particular, there are 954 fully disordered sequences for all 10,000 natural sequences but only 139 for random sequences with natural amino acid compositions and 0 for random sequences with fixed amino acid compositions.

While there is a large difference in three sequence databases for number of proteins with long disordered regions (Fig. 2a), the difference is not significant for number of proteins (within standard deviations) with long structured regions (>50 residues, Fig. 2b). Random sequences tend to have more sequences with structured regions between 40 and 50 residues. There are 58 proteins with ≥55 residues in a continuous structured region for Pnat, 55 for Prnd, and 24 for Preq. The small difference between 58 for natural sequences and 55 for random sequences with the same overall composition of amino acids suggests that natural sequences are only slightly or marginally more optimized than random sequences for full structured proteins.

To confirm the accuracy of predicted structured and disordered regions (defined by SPINE-D with a threshold at 0.5), we investigated composition bias ($\Delta P_{\text{i}}^{\text{o}}$ and $\Delta P_{\text{i}}^{\text{d}}$) in structured and intrinsically disordered regions and compared to annotated regions in the DisProt database [29]. Composition bias in predicted regions (ordered or disordered) by SPINE-D is highly similar to that in annotated regions for three separate sequence databases with high pairwise Pearson’s correlation coefficients. For structured regions, the correlation coefficients to annotated regions are 0.75 for natural sequences, 0.91 for Prnd, and 0.90 for Preq, respectively. For intrinsically disordered regions, the correlation coefficients to annotated regions are 0.74 for natural sequences, 0.90 for Prnd, and 0.90 for Preq, respectively. Lower correlation coefficients of composition biases between natural sequences and annotated regions are likely because composition biases in random sequences play more important roles in disorder classification as a result of less informative sequence profiles from multiple sequence alignment than natural sequences.

Secondary structural contents predicted by SPIDER2 for three sequence datasets in structured and disordered regions are compared in Table 1. The difference is small but statistically significant (*p* value for unpaired *t* test <0.002 for all cases): Pnat has 3–7 % higher fraction of helical residues per protein (35.6 %) than Prnd (32.9 %) and Preq (28.3 %) but 7 % less sheet residues (22.6 %, compared to 29.9 % for Prnd and 29.4 % for Preq) in the structured regions. All sequences in disordered regions have significantly (10 % or more) less helical and sheet residues than in structured regions. Table 1 also tabulated fractions of annotated helical and sheet residues in 110 non-redundant monomeric protein structures (Pstruc). Helical and sheet contents in Pstruc are similar to those in Pnat, confirming the overall accuracy of predicted secondary structures.

**Table 1:** The average helical and sheet contents in structured and disordered regions

Database	Structured		Disordered	
	Helix	Sheet	Helix	Sheet
Preq^a^	0.28 ± 0.20	0.29 ± 0.17	0.15 ± 0.17	0.10 ± 0.12
Prnd^a^	0.33 ± 0.24	0.30 ± 0.19	0.20 ± 0.19	0.12 ± 0.13
Pnat^a^	0.36 ± 0.29	0.23 ± 0.22	0.21 ± 0.19	0.09 ± 0.11
Pstruc^b^	0.37 ± 0.32	0.21 ± 0.18	−	−

^a^Based on predicted secondary structure by SPINE-D

^b^Based on actual secondary structure by DSSP

**Fig. 3:**
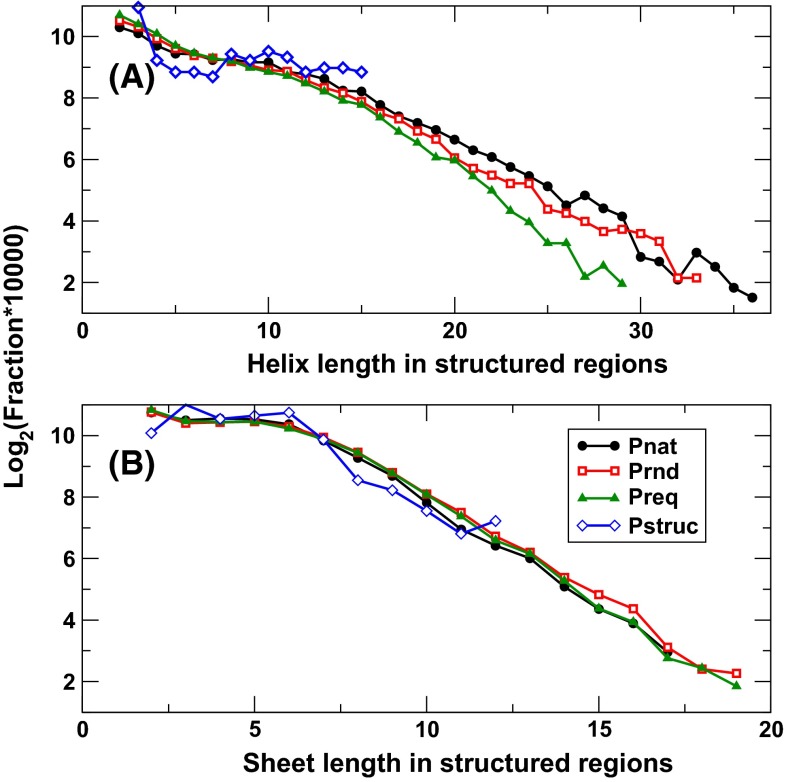
The fraction of helices (**a**) and sheets (**b**) in a given length [log_2_ (fraction × 10,000)] in structured regions for four databases as labeled. To ensure statistics, the sizes of helices or sheets that appeared in less than five proteins in the dataset are not shown

Figure 3 compares the length distribution of helices and sheets in Preq, Prnd, Pnat, and Pstruc in structured regions. The difference between Pnat and Prnd is small. This indicates that natural and random sequences (given the same overall compositions) have similar helical and sheet lengths. Similar distribution is observed for structured proteins (Pstruc) although the dataset is much smaller (110 vs. 10,000 sequences), suggesting that there is no evolutionary preference in lengths of helices and sheets in protein structures.

**Fig. 4:**
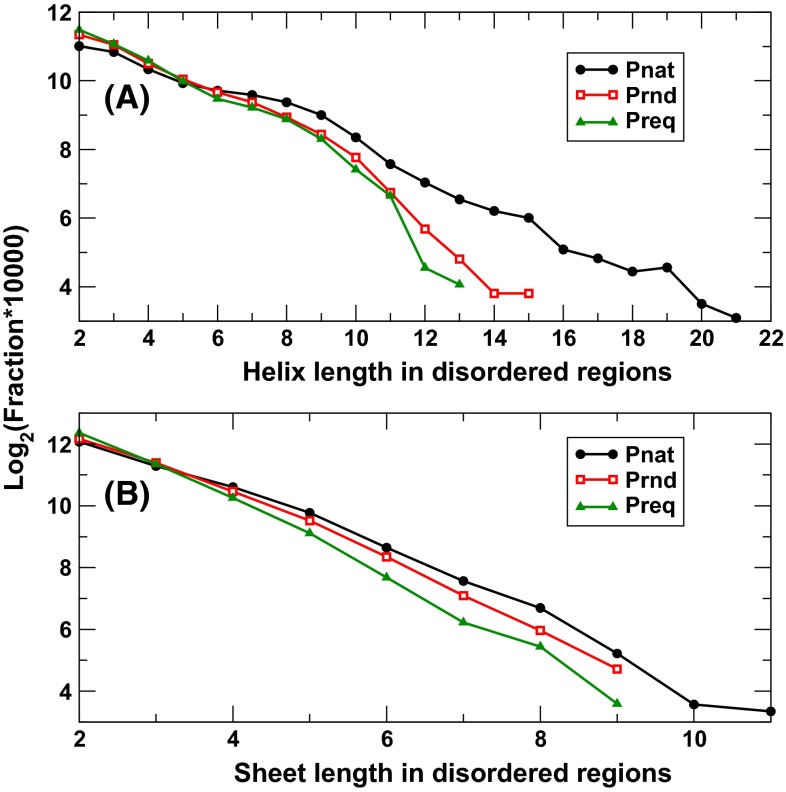
The fraction of helices (**a**) and sheets (**b**) in a given length [log_2_ (fraction × 10,000)] in intrinsically disordered regions for three databases as labeled

Figure 4 compares the length distribution of helices and sheets in Preq, Prnd, and Pnat in intrinsically disordered regions. Pnat has more long helices than Prnd and Preq. This is largely because Pnat has significantly more long continuously disordered regions (Fig. 2). However, the length distributions of sheets are much closer to each other, despite that Pnat has more proteins with long disordered regions.

Can random sequences have well-defined three-dimensional structures? We performed the fold recognition method SPARKS X [28] for all proteins with predicted structural regions of more than 54 residues (59 for Pnat, 55 for Prnd and 24 for Preq). SPARKS X is a method that attempts to map a query sequence of unknown structure to all known structures stored in the protein databank based on multi-dimensional matches of sequence and structural information. The significance of a match is measured by a *Z*-score with *Z*-score >7 suggesting a highly significant match. There are 25 out of 59 proteins with *Z*-score >7 for Pnat, two out of 55 for Prnd, and three out of 24 for Preq. Despite a similar number of proteins with predicted structural regions of more than 54 residues, Pnat has many more predicted proteins with quality predicted structures than Prnd. This is largely because natural sequences have more naturally occurring homologs or remote homologs.

**Fig. 5:**
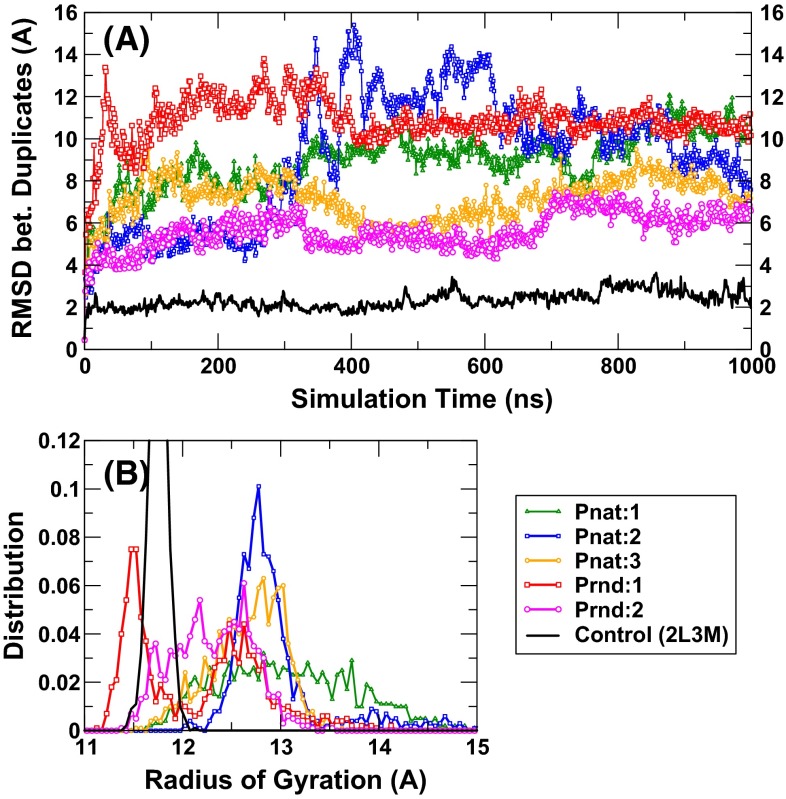
**a** The RMSD between two conformations from two independent simulations as a function of simulation time for six proteins (Pnat: 1, 2, and 3 refer to UniRef50_M0WDE6, UniRef50_J9E0E9, and UniRef50_D6GUH9, respectively; Prnd: 1 and 2 refer to seq 08789 and seq 04514, respectively). **b** The distribution of radius of gyration for the last 50 ns of two duplicate simulations for each sequence

**Fig. 6:**
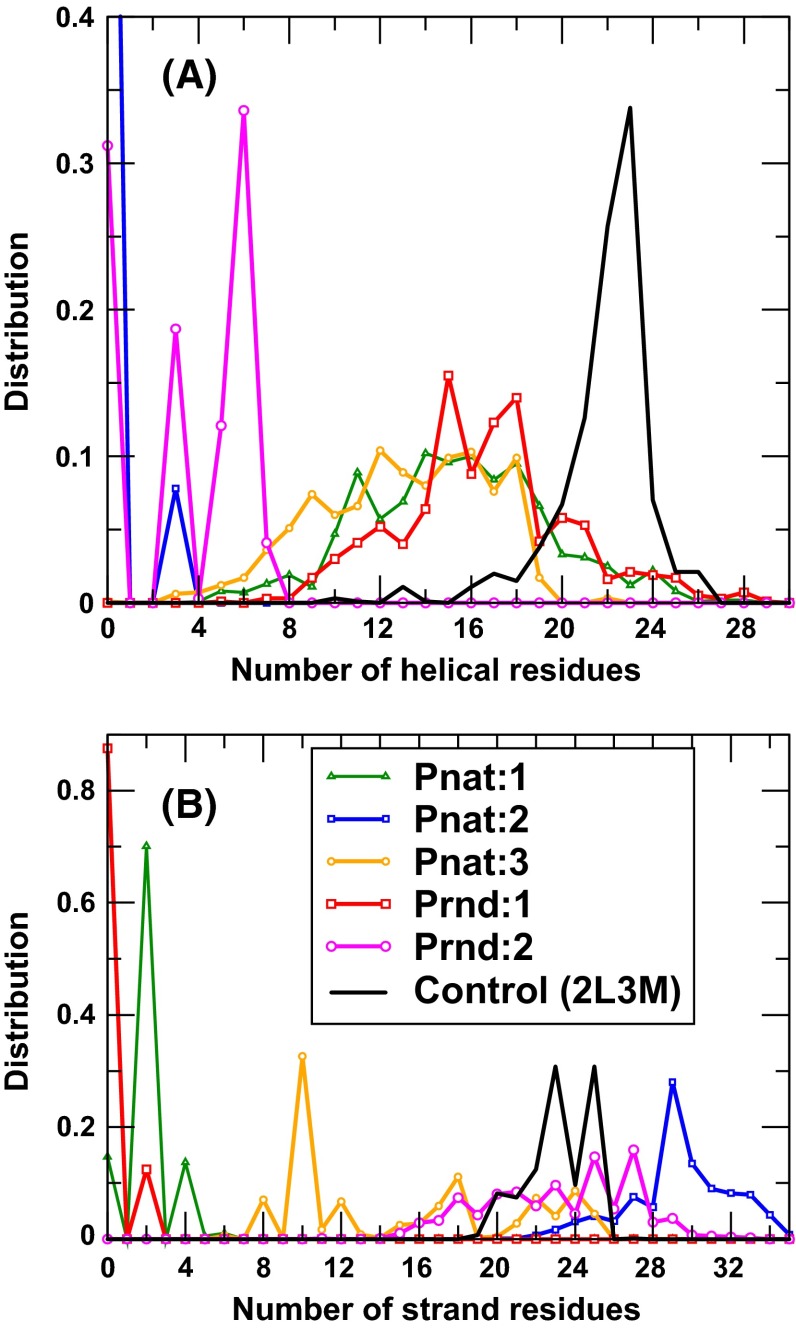
**a** The distribution of the number of helical residues for the last 50 ns of two duplicate simulations for each of six proteins (Pnat: 1, 2, and 3 refer to UniRef50_M0WDE6, UniRef50_J9E0E9, and UniRef50_D6GUH9, respectively; Prnd: 1 and 2 refer to seq 08789 and seq 04514, respectively). **b** As in (a) but for the number of strand residues

We performed molecular dynamics simulations for two sequences from Prnd (Seq 08789 and Seq 04514 with *Z*-score = 7.41 and 7.07, respectively) and three sequences from Pnat (UniRef50_M0WDE6, UniRef50_J9E0E9, and UniRef50_D6GUH9 with *Z*-score = 9.24, 8.97, and 8.94, respectively). As a control, we also performed MD for one solution NMR structure of a putative copper-ion-binding protein from *Bacillus anthracis* str. Ames (PDB ID 2L3 M, 71 residues long). All models either from Pnat or from Prnd failed to have a stable structure after 100-ns simulations (6–10 Å RMSD from the starting conformations and 7–10 Å between two last conformations in duplicate simulations, Fig. 5a) while the PDB structure 2L3 M remains stable (2.8 and 2.6 Å RMSD, respectively, from the native conformation) after 100-ns simulation. Interestingly, only minor increases in radius of gyration were observed for Prnd (1 and 6 %) and Pnat sequences (−2, 3, and −6 %, respectively). The distributions of radius of gyration in the last 50 ns for all six pairs of simulations are shown in Fig. 5b. These results indicate that model structures are more flexible and slightly less compact than the native structure (2L3 M). Figure 6 further examines the distribution of amount of secondary structures (helical and sheet residues in Fig. 6a, b, respectively) in model structures as compared to the native structure (2L3 M). It is clear that the distributions of the numbers of helical and sheet residues are much narrower in native structures than in model structures. These results indicate that model structures are not accurate enough to confirm whether random sequences are capable of having unique structures by molecular dynamics simulations. Nevertheless, MD simulation results confirm that random sequences are capable of forming collapsed globule structures with some secondary structures.

## Discussion

We have studied structure and disorder in 10,000 naturally occurring and random protein sequences by using current state-of-the-art techniques for prediction of protein intrinsic disorder, secondary structure, and tertiary structure. Based on intrinsic disorder prediction, natural sequences have many more disordered residues in long continuous regions but only marginally more nearly full-structured proteins than random sequences. In predicted structured regions, natural sequences have marginally higher helical residues but less sheet residues than random sequences with the same amino acid compositions. In predicted disordered regions, there is no significant difference in helical and sheet contents between natural and random sequences of the same amino acid compositions. The distributions of helical and sheet lengths for random and natural sequences follow essentially the same power-law distribution in the structured region. Although molecular dynamics simulations of a few selected model structures did not reveal stable conformations, these model structures remain highly compact, suggesting that these proteins (with random and natural sequences) at least are collapsed molten globules with some secondary structures.

Random protein sequences are nearly as structured or more structured than natural sequences. This finding, based on disorder prediction and prediction of secondary structure, is consistent with several experimental examinations of sequences from random co-polymerization of mixed amino-acid* N*-carboxyanhydrides [6, 7], random three residue types (Q, R, and L) of 70–90 amino acid residues [8], random 120-amino-acid sequences of 20 and 12 residue types [9], random 50-residue proteins [10], and random 71-residue proteins [11]. These experimental studies showed that random sequences have compact structures, cooperative unfolding, secondary structures, and/or protected from serine protease thrombin. The consistency between experimental and our computational studies occurs despite that experimental protein sequences were obtained at DNA levels, expressed in *E. coli* (i.e., subjected to codon optimization).

It should be noted, however, that SPINE-D [26] likely over-predicts structured regions because it cannot distinguish proteins in molten globule states (compact with some secondary structures [30]) from proteins in unique three-dimensional structures. This happens because only native structures and disordered regions were employed for training SPINE-D [26]. Indeed, long molecular dynamics simulations of predicted model structures of random sequences failed to produce a well-defined conformation. However, model structures of natural sequences also failed to have a well-defined conformation, suggesting that model inaccuracy is likely the main reason for unfolding of model structures in molecular dynamics simulations. If the majority of predicted structured regions are in a molten globule state, it explains the difficulty in producing folded structures from in vitro random recombination of secondary structure elements (blocks) [14, 15].

What is interesting is that natural sequences have more disordered residues and more long disordered regions with helical conformations. In a recent paper, we have shown that the fraction of order and semi-disorder (disorder probability <0.7) predicted by SPINE-D can be effectively employed to predict residues in aggregation prone regions with an accuracy comparable to several state-of-the-art techniques dedicated for aggregation prediction [31]. Thus, more disordered and long disordered regions for natural sequences indicate that natural sequences are created and evolved for solubility so as to avoid protein aggregation. This is consistent with the finding that random 120-amino-acid sequences of 20 residue types are aggregation-prone [9]. The existence of helical regions in long disordered regions indicates that nature may also employ disorder to enhance plasticity for function because helices in disordered regions are one of the widely utilized motifs in protein–protein interactions [32]. Disordered regions also provide accessibility of key residues for post-translational modifications, and serve as flexible linkers for separating functional domains or entropic bristles for keeping non-interacting molecules apart [33].

## Materials and methods

### Construction of protein sequence databases

Natural sequences in Pnat are obtained from the UniRef50 sequence database [34]. Its sequence redundancy was removed by using BLASTClust [35] with 30 % sequence identity cut-off. Sequence non-redundancy in Prnd and Preq was examined and confirmed by the program CD-HIT [36] with 30 % sequence identity cut-off. The natural occurrences of amino acid types were obtained from BLOSUM62 [37].

### Intrinsic disorder prediction

The existence of intrinsic disorder in proteins (natural or artificial sequences) is probed by two different algorithms. One method is IUpred, which predicts disorder based on knowledge-based interaction strengths within sequentially neighboring amino acid residues [25]. IUpred is computationally fast because it does not require evolutionary information of protein sequences. Another method is SPINE-D, which employs a neural network trained for disorder prediction [26]. SPINE-D provides a more accurate prediction of intrinsic disorder than IUpred and was independently assessed to be among the best-performing methods in the Critical Assessment of Structure Prediction techniques (CASP 9, 2010) [38]. It is more accurate because protein evolutionary information accounts for the fact that structured regions are more likely conserved than unstructured, intrinsically disordered regions. Comparing predictions between IUpred and SPINE-D will allow us to evaluate the consistency in computational predictions in the presence and absence of sequence evolution information.

### Protein secondary-structure prediction

A recently developed method SPIDER2 [27] was employed to predict secondary structure by iterative deep learning of multiple structural properties (backbone torsion angle, solvent accessible surface area, and Calpha angles) in addition of secondary structure. It was chosen because it is one of the most accurate predictors of secondary structures.

### Protein secondary structure analysis

For comparison, we also obtained structured proteins (Pstruc) with 3.5-Å resolution or better and sequence lengths between 50 and 70 amino acid residues from the protein databank. We further removed protein structures that are in complex with RNA, DNA, or proteins. The final dataset (Pstruc) contains 110 proteins after removing redundancy at 30 % sequence-identity cut-off. The secondary structures of these proteins were obtained from the PDBfinder database [39]. Eight-state annotations were merged into three states [H, G, and I for Helix (H), B and E for sheet (E), T, S, and D for Coil (C)].

### Amino acid preferences

We evaluated the preferences of amino acid residues in ordered or intrinsically disordered regions by examining the difference of their occurrence in the region $(P_{\text{i}}^{\text{o}} ,P_{\text{i}}^{\text{d}} )$ from their occurrence in all sequences in the database $(P_{\text{i}}^{\text{all}} )$ [40]. That is, $\Delta P_{\text{i}}^{\text{o}} = (P_{\text{i}}^{\text{o}} - P_{\text{i}}^{\text{all}} )/P_{\text{i}}^{\text{all}}$ and $\Delta P_{\text{i}}^{\text{d}} = (P_{\text{i}}^{\text{d}} - P_{\text{i}}^{\text{all}} )/P_{\text{i}}^{\text{all}}$ in addition to calculating amino acid preferences from predicted ordered and disordered regions, we also calculated amino acid preferences in annotated structured and disordered regions by using the DisProt database [29]. A total of 548 annotated sequences were obtained from the DisProt database after removing redundancy by using CD-HIT (30 % sequence identity cut-off). These sequences contain 911 intrinsically disordered regions and 978 structured regions.

### Structure prediction and molecular dynamics simulations

For those random sequences predicted to be structured, we performed template-based structure prediction by SPARKS X with default parameters [28]. Selected model structures are then simulated in the presence of water molecules. Molecular dynamics (MD) simulation in the isothermal-isobaric (NPT) ensemble was performed using the GROMACS 4.6.2 software package [41]. We employed the amber99sb-ildn force field for proteins and TIP3P for water molecules [42]. The protein was solvated in a truncated octahedron box with the minimum solute-box boundary distance being set to 12 Å. The long-range electrostatic interaction was treated with the particle-mesh Ewald method with a grid spacing of 1.2 Å and a fourth-order interpolation [43, 44]. Protonation states of ionizable groups were chosen for pH = 7.0. For each protein, two independent simulations were performed for 100 ns with different initial velocities for pressure *P* at 1 bar and temperature *T* at 298K. The temperature of the system was kept constant by velocity rescaling with a stochastic term [45]. The pressure of the system was kept constant by using the Berendsen algorithm [46]. The simulation employed a temperature coupling time of 0.1 ps and pressure coupling time of 2 ps. The time step for the MD integrator was set to 2 fs and LINCS [47] was applied to constrain all bond lengths.

### Availability of data and materials

All sequence datasets (Pnat, Prnd, and Preq) are made available at http://sparks-lab.org.
